# Pennsylvania State Dental Society

**Published:** 1895-07

**Authors:** 


### PENNSYLVANIA STATE DENTAL SOCIETY.

We are requested to call special attention to this meeting, to take place at Eagles' Mere, Pennsylvania, July 9, 10, and 11.

The dental profession in this State should make an unusual effort to have this a gathering of great interest. The programme includes a series of papers of unusual promise, and it only needs the encouragement of a large attendance to bring this convention up to the vigor of earlier years. There will be a large delegation of eastern members in attendance. It is to be regretted that our colleagues in the western part of the State will have difficulty in reaching the place selected. It is certainly not a convenient location, however beautiful it may be, and it is hoped that a locality more easily accessible may be fixed upon for the meeting next year, 1896, and the years to follow.

